# Subcellular localization of SARS-CoV-2 E and 3a proteins along the secretory pathway

**DOI:** 10.1007/s10735-025-10375-w

**Published:** 2025-03-01

**Authors:** Joshua J. Hinkle, Kathleen A. Trychta, Emily S. Wires, Raven M. Osborn, Justin R. Leach, Zoha F. Faraz, Reinis Svarcbahs, Christopher T. Richie, Stephen Dewhurst, Brandon K. Harvey

**Affiliations:** 1 Intramural Research Program, National Institute on Drug Abuse, NIH, Suite 200, 251 Bayview Blvd, Baltimore, MD 21224 USA; 2 School of Medicine & Dentistry, University of Rochester, Rochester, NY 14642 USA

**Keywords:** SARS-CoV-2, E protein, 3a protein, Secretory pathway, Subcellular localization

## Abstract

SARS-CoV-2 E and 3a proteins are important for the assembly, budding, and release of viral particles. These two transmembrane proteins have been implicated in forming channels in the membrane that allow the transport of ions to favor viral replication. During an active infection, both proteins generally localize to the endoplasmic reticulum (ER), ER-Golgi intermediate compartment (ERGIC), and the Golgi where viral assembly occurs. The ER and Golgi are critical for the proper packaging and trafficking of cellular proteins along the secretory pathways which determine a protein’s final destination inside or outside of the cell. The SARS-CoV-2 virus primarily infects epithelial cells that are highly secretory in nature such as those in the lung and gut. Here we quantified the distribution of SARS-CoV-2 E and 3a proteins along the secretory pathways in a human intestinal epithelial cell line. We used NaturePatternMatch to demonstrate that epitope-tagged E and 3a proteins expressed alone via transient transfection have a similar immunoreactivity pattern as E and 3a proteins expressed by wild-type viral infection. While E and 3a proteins localized with all selected cellular markers to varying degrees, 3a protein displayed a higher correlation coefficient with the Golgi, early/late endosome, lysosome, and plasma membrane when compared to E protein. This work is the first to provide quantification of the subcellular distribution of E and 3a proteins along the multiple components of the secretory pathway and serves as a basis to develop models for examining how E and 3a alter proteostasis within these structures and affect their function.

**Supplementary Information:**

The online version contains supplementary material available at 10.1007/s10735-025-10375-w.

## Introduction

The severe acute respiratory syndrome coronavirus-2 (SARS-CoV-2), responsible for the worldwide pandemic coronavirus disease 2019 (COVID-19), is related to the SARS-CoV virus strain that caused the 2003 outbreak (Yoshimoto 2020; Abdolmaleki et al. 2022). The recurrence of coronavirus outbreaks indicates their propensity to evolve, increasing the likelihood for future outbreaks. Despite sequence homology and similarities between SARS-CoV proteins it took several years to approve mitigative treatments for individuals following symptomatic SARS-CoV-2 infection (Murakami et al. 2023; Arevalo-Romero et al. 2024). For this reason, it is important to investigate commonalities among coronavirus strains for panviral therapeutic strategies to combat respiratory distress and reduce fatality in infected individuals. Accumulating evidence indicates that persistent ER calcium dysregulation and ER stress is associated with many disorders, such as neurodegenerative diseases, cardiovascular disease, immune and inflammatory diseases, diabetes, cancer, and viral infection (Mekahli et al. 2011; Zhai et al. 2020; Ghemrawi and Khair 2020). By developing new therapeutics or repurposing FDA-approved drugs, pathways that are modulated by the virus can be targeted to reduce virulence and detrimental host immune reactivity; specifically, mechanisms involved in endoplasmic reticulum (ER) homeostasis, proteostasis, and calcium gradient regulation (Fung and Liu 2019; Li et al. 2019; Jiang et al. 2020).

SARS-CoV-2 is an enveloped, single-stranded, positive-sense RNA virus that encodes 11 protein coding open reading frames (ORFs) that cleave into structural, accessory, and non-structural proteins that aid in the viral life cycle (Gordon et al. 2020; Yoshimoto 2020; Tebha et al. 2024). The viral genome invades the host cell cytoplasm via Spike (S) protein-mediated angiotensin-converting enzyme 2 (ACE2) receptor binding, and once internalized, the positive-sense RNA genome is translated and its protein products are trafficked to form protein complexes at the ER membrane and ER-Golgi intermediate compartment (ERGIC). These complexes can affect protein synthesis, packaging, and distribution by hijacking the secretory pathway to favor viral replication as well as to evade host detection before egress from the host cell (Fung and Liu 2019; Hartenian et al. 2020; Mukherjee et al. 2020; Aoe 2020).

Viral channel forming proteins, herein referred to as viroporins, are expressed by several viruses and typically form homo-oligomeric, hydrophilic pores that embed into host cellular membranes and allow ions, such as calcium to transit such membranes (Nieva et al. 2012; Hyser and Estes 2015; Scott and Griffin 2015; Breitinger et al. 2022). Depending on the stage in the viral life cycle, viroporins can induce or inhibit apoptosis by exploiting calcium signaling pathways (Hyser and Estes 2015). Some viroporins that localize to the ER cause alterations in pH in the Golgi network to modulate protein trafficking (influenza A virus, M2 protein; Cady et al. 2011). Previous studies suggested that SARS-CoV viral proteins E and 3a (ORF3a) act as viroporins to provoke ion imbalance, including changes in calcium, and disrupt cellular pathways to aid replication and/or virulence (DeDiego et al. 2014; Nieto-Torres et al. 2015). Due to the high sequence homology between SARS-CoV and -CoV-2 E and 3a proteins, similar functions have been attributed to these proteins in SARS-CoV-2 (E: Breitinger et al. 2023, Harrison et al. 2022; 3a: Kern et al. 2021, Fam et al. 2023); however, recent studies suggest that 3a protein is not a viroporin (McClenaghan et al. 2020; Jiao et al. 2023; Miller et al. 2023; Oliveira-Mendes et al. 2023).

The E protein is an 8.4–12 kDa membrane protein with 94.7% sequence identity between SARS-CoV and -CoV-2 (Yoshimoto 2020). The E protein is integral to viral assembly, virion release, and virulence (McClenaghan et al. 2020; Schoeman and Fielding 2019) and E-deletion mutants have 100- to 1000- fold lower titers than wild-type virus in the respiratory tract of hamsters (as well as in cultured cell lines; DeDiego et al. 2007). Only a small proportion of E protein is incorporated into the virion while the majority of the E protein accumulates along the secretory pathway in the ER, ERGIC, Golgi, endosomes, and lysosomes (Boson et al. 2021; Henke et al. 2022; Miserey-Lenkei et al. 2021; Neitthoffer et al. 2024; Miura et al. 2023) where it interacts with other viral proteins to aid in the assembly, budding, and release of viral particles (Fung and Liu 2019; Hartenian et al. 2020).

SARS-CoV-2 ORF3a protein (31 kDa) shares 72.4% sequence identity between SARS-CoV and -CoV-2 (Yoshimoto 2020). Although SARS-CoV-2 3a protein is more closely related to Bat coronaviruses than SARS-CoV (91% sequence identity to Bat-SL-CoV; Grifoni et al. 2020), conserved critical regions suggest that the 3a protein has similar molecular functions (Chan et al. 2009; McClenaghan et al. 2020). The 3a protein is important for viral packaging and release, and to a lesser extent, replication, as demonstrated in several models (Chan et al. 2009; Freundt et al. 2010; Castano-Rodriguez et al. 2018; Siu et al. 2019). The 3a protein has been localized to the Golgi apparatus, endosomes, lysosomes, and plasma membrane (Miller et al. 2023; Zhang et al. 2020; Cruz-Cosme et al. 2022; Lee et al. 2021; Miserey-Lenkei et al. 2021) but the expression level and localization vary among studies.

Identifying the subcellular distribution and function of SARS-CoV-2 E and 3a proteins in relation to protein trafficking and secretion is needed to understand their potential to alter cellular proteostasis and health. The ER plays a crucial role in protein quality control, as protein folding is monitored and governed by an array of ER resident, calcium-dependent chaperones. These ER resident chaperones bind unfolded or misfolded proteins until they are properly folded or transport them for proteasomal or lysosomal degradation (Sala et al. 2017). Several studies have demonstrated that depletion of ER calcium stores and subsequent disruption to calcium homeostasis can lead to the secretion of ER resident proteins from the cell that are crucial for the cell’s survival (Mekahli et al. 2011; Trychta et al. 2018; Henderson et al. 2021). Additionally, adaptive responses can be activated by ion gradient dysregulation and misfolded proteins, including the unfolded protein response (UPR; Hetz et al. 2020), autophagy (Bootman et al. 2018), and apoptosis (Sukumaran et al. 2021). Collectively, E and 3a proteins may alter ion balances, influence calcium channel coordination and pH levels, and activate the UPR. Here, the purpose was to examine the subcellular localization of SARS-CoV-2 proteins E and 3a relative to the organelles along the secretory pathway and the effects of each protein on UPR activation.

## Results

### SARS-CoV2 E and 3a proteins are multimeric, transmembrane proteins conserved across multiple SARS-CoV-2 variants

**Fig. 1:**
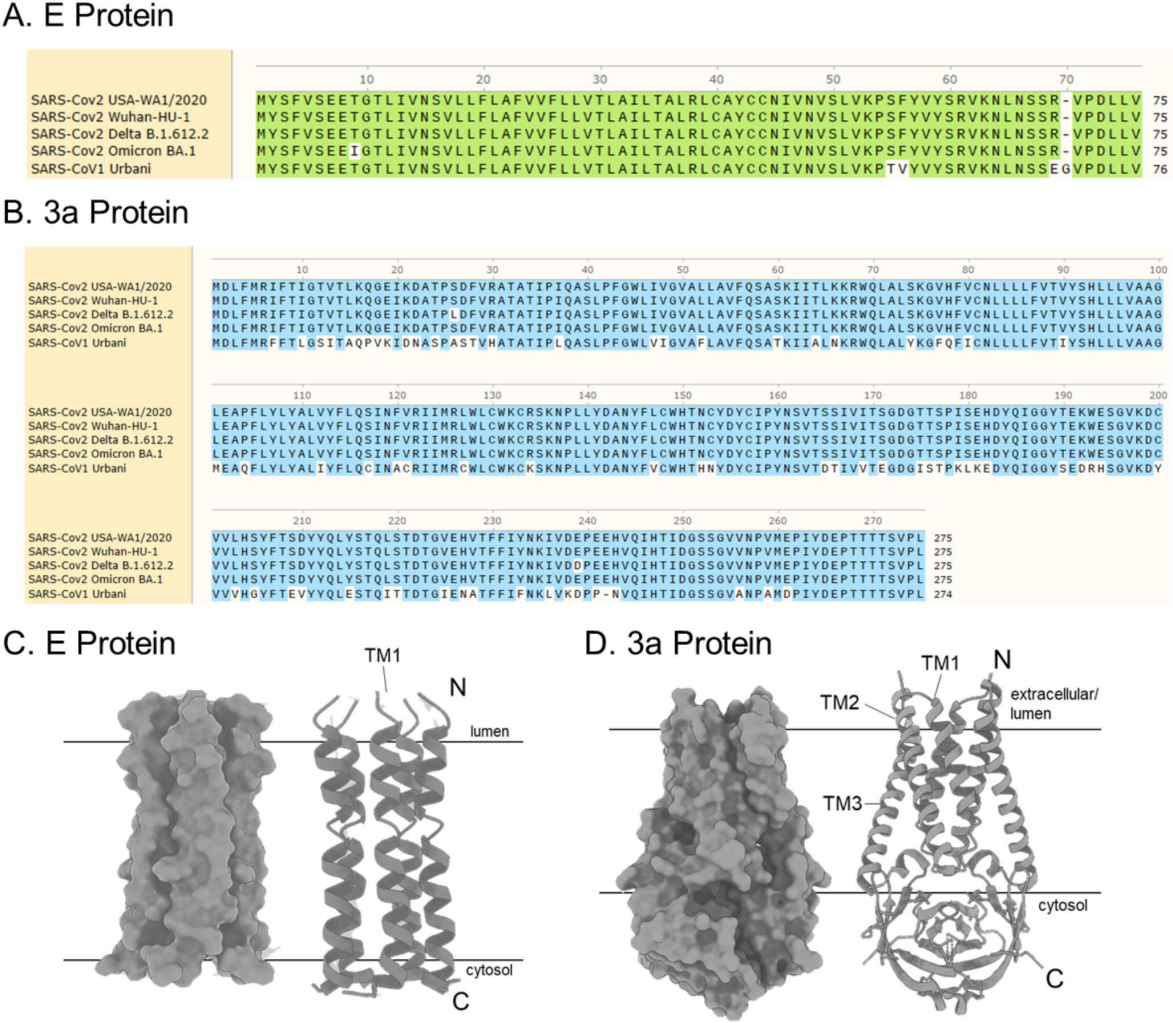
SARS-CoV-2 proteins E and 3a are highly conserved across variants. Protein sequence alignment of (**A**) Envelope and (**B**) 3a (ORF3a) protein from SARS-CoV Urbani (AY278741) and SARS-CoV-2 variants: Wuhan-HU-1 19 A (MN908947), USA-WA1/2020 (MN985325.1), Delta B.1.612.2 21 A (MZ009823), Omicron BA.1 21 K (prev. B.1.1.529; OL672836). Schematic of (**C**) pentameric Envelope protein (Mandala et al. 2020; Medeiros-Silva et al. 2022) and (**D**) dimeric 3a protein (Kern et al. 2021) structure and helix with proposed transmembrane orientation (generated in UCSF ChimeraX)

The E protein is a single pass transmembrane protein shown to form a pentameric complex (Medeiros-Silva et al. 2022, 2023; Fig. 1A). The 3a protein is a three-pass transmembrane protein forming a multimeric complex (Kern et al. 2021; Fig. 1A). Protein sequence alignment demonstrates high conservation of E protein and 3a protein from SARS-Cov-2 variants suggesting similar structure and potentially function across variants (Fig. 1A). Protein sequences were taken from GenBank and aligned for SARS-CoV Urbani (AY278741) and SARS-CoV-2 variants: Wuhan-HU-1 19 A (MN908947), USA-WA1/2020 (MN985325.1), Delta B.1.612.2 21 A (MZ009823), Omicron BA.1 21 K (prev. B.1.1.529; OL672836).

### SARS-CoV-2 USA-WA1/2020 E and 3a protein immunofluorescence correlates with cellular markers of the ER and Golgi in viral infected cells

**Fig. 2:**
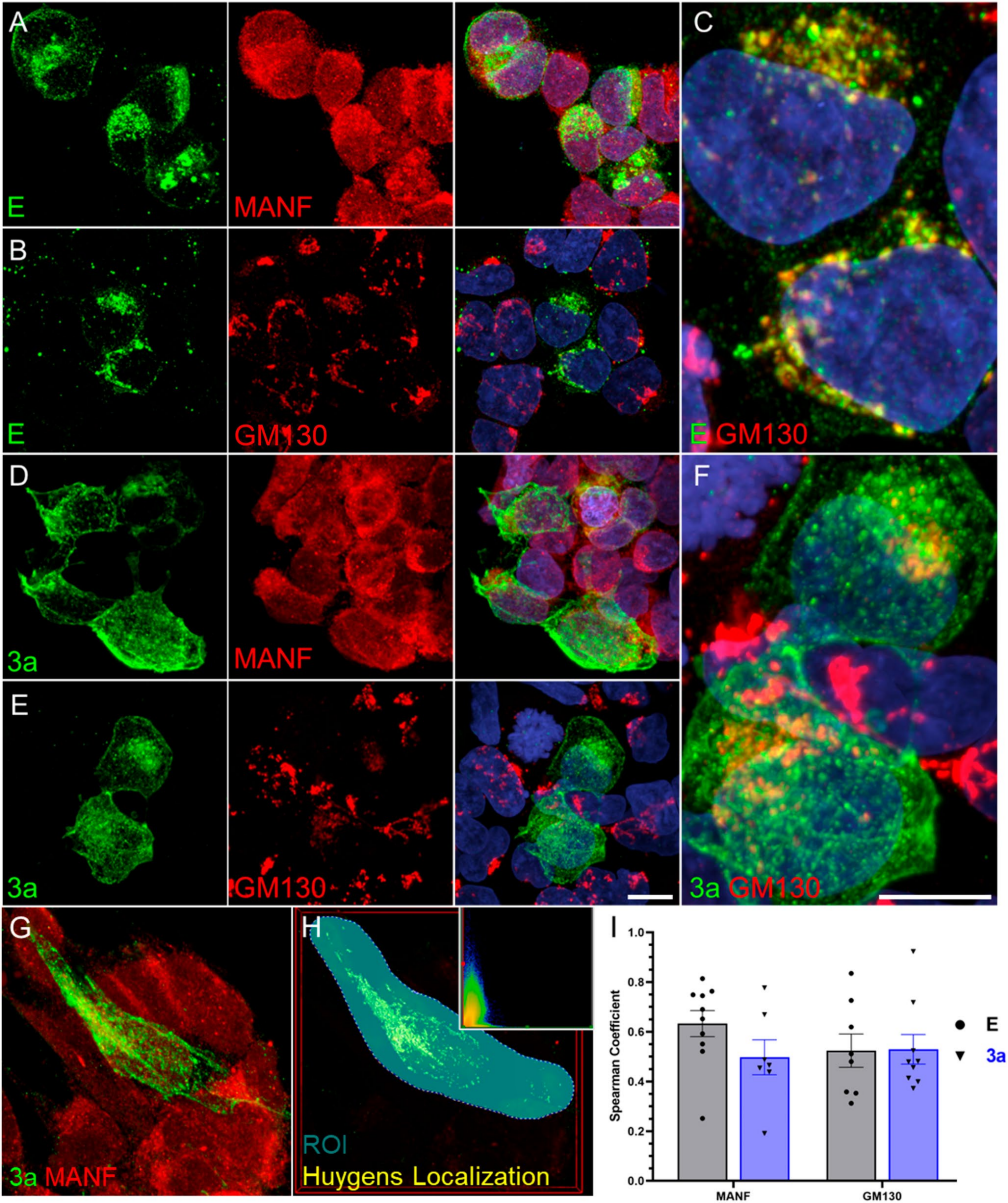
Representative confocal maximum intensity projected z-stack images of SARS-CoV-2 USA-WA1/2020 infected Caco-2 cells 8 h post-infection. **A**, **B**, **C**) **E** protein and (**D**, **E**, **F**) 3a protein staining with organelle markers MANF and GM130 demonstrating localization patterns. Zoomed insets demonstrating GM130 localization with (**C**) E protein and (**F**) 3a protein. **G**) Input image of 3a protein and GM130 for (**H**) Huygens Spearman Correlation ROI analysis with inset example scatter plot of organelle marker (red) vs. pixel value of viral protein (green). **I**) Quantitative Spearman correlation of E and 3a protein with MANF and GM130. **A**-**F**) Scale bar: 20 μm. Unpaired, two-tailed t-test comparison, *p* > 0.05

To visualize and quantify E and 3a protein expression following a wild-type viral infection, Caco-2 cells were infected with SARS-CoV-2 Washington variant and localization with both MANF (mesencephalic astrocyte-derived neurotrophic factor, ER marker; Spearman Correlation mean: E – 0.63, 3a – 0.50) and GM130 (*cis*-Golgi; mean: E – 0.52, 3a – 0.53) were found. This suggests that E and 3a proteins have similar distribution in the ER and Golgi (Fig. 2I). However, qualitatively, while both proteins had vesicle-like puncta staining the E protein appeared more ER-like and 3a protein had more apparent plasma membrane-like staining (Fig. 2A-F; Supplemental Fig. 2).

### The subcellular pattern of immunoreactivity for SARS-CoV2 USA-WA1/2020 E and 3a proteins is similar between viral infected cells and transiently transfected cells

**Fig. 3:**
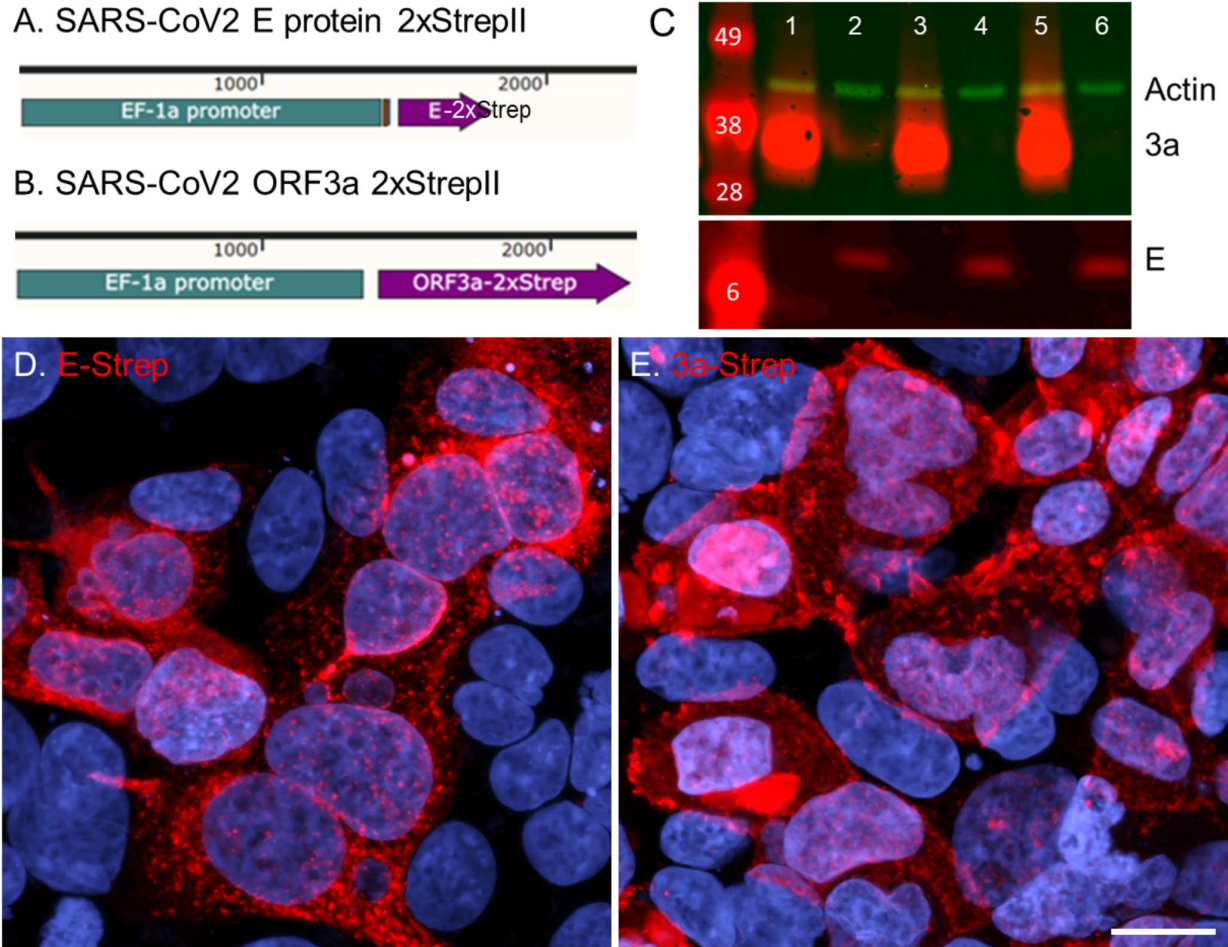
SARS-CoV-2 E and 3a 2xStrepII proteins demonstrate different staining patterns. Plasmid map for SARS-CoV-2 (**A**) E protein and (**B**) 3a protein with 2xStrepII tag (-Strep). **C**) Western blot of Strep tagged E (lanes 2, 4, 6) and 3a (lanes 1, 3, 5) transfected Caco-2 cells. Representative confocal maximum intensity projected z-stack images of transfected SARS-CoV-2 (**D**) E and (**E**) 3a protein plasmids in Caco-2 cells stained with Strep antibody and DAPI. **D**, **E**) Scale bar: 20 μm

**Fig. 4:**
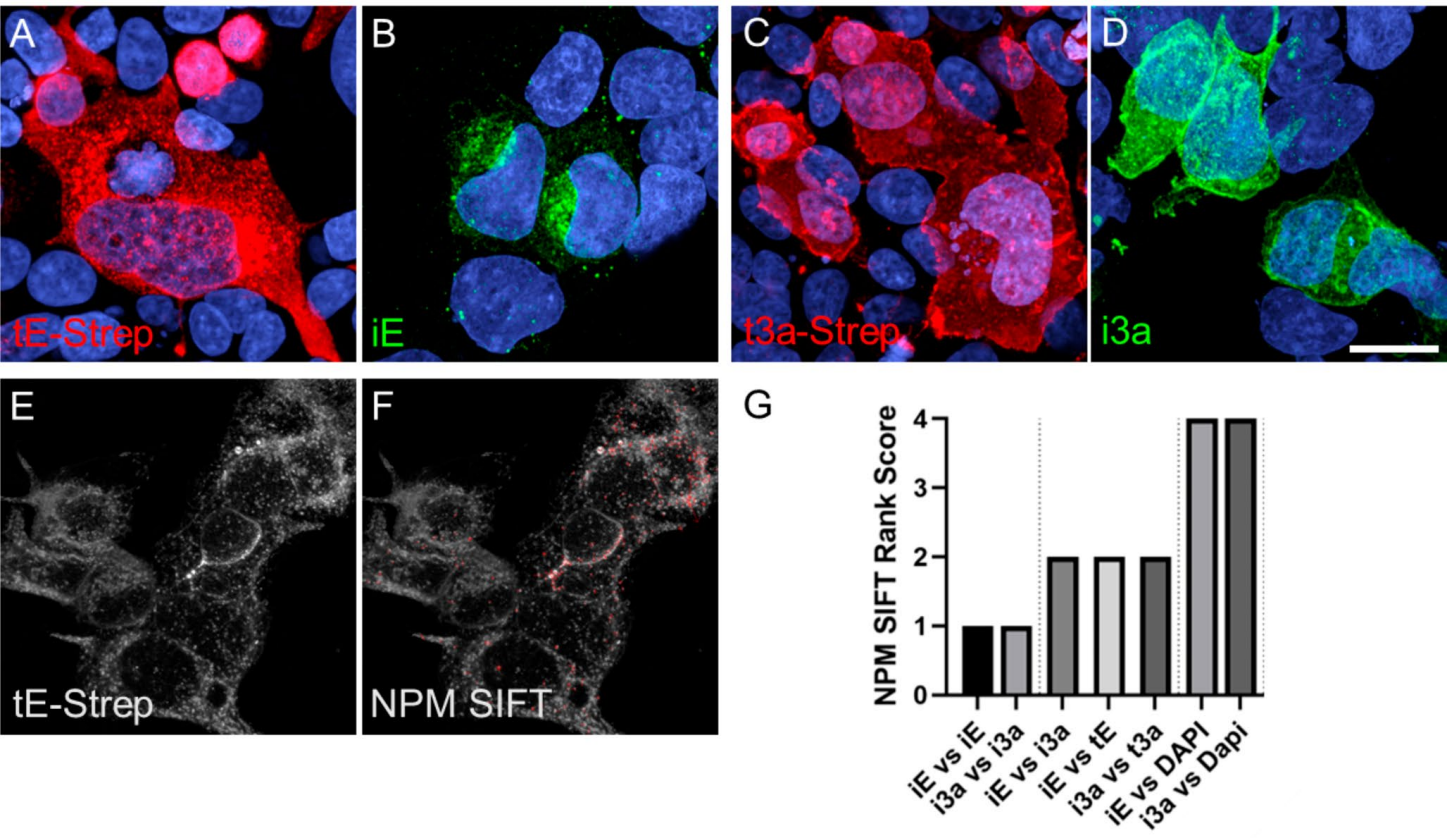
Representative confocal maximum intensity projected z-stack images of SARS-CoV-2 proteins and subsequent pattern matching algorithm. Comparison of (**A**) transfected and (**B**) infected E protein and (**C**) transfected and (**D**) infected 3a protein in Caco-2 cells. **E**) Example image of original NaturePatternMatch (NPM) input image and (**F**) SIFT keypoint pattern extraction output. **G**) Quantitative rank scores of pattern similarities generated from NaturePatternMatch SIFT feature extraction image analysis (1 – highly similar, 4 – no similarity; i = infection, t = transfection). **A**-**F**) Scale bar: 20 μm

To study the subcellular localization of E and 3a without the need for BSL3 (Biosafety Level 3) procedures, plasmids were engineered and verified via western blot (Fig. 3C) and immunocytochemistry (Fig. 3D, E) to transiently express E or 3a protein with 2xStrep tag via transfection (Supplemental Fig. 1). Visually, the immunoreactivity pattern for viral infected E (iE; Washington variant) and the pattern of transiently transfected E (tE: plasmid) appeared similar in Caco-2 cells (Fig. 4A, B). Infected 3a (i3a) and transient transfected 3a (t3a) proteins both had plasma membrane-like immunoreactivity but differed in overall membrane patterns (flattened versus rounded; Fig. 4C, D, Supplemental Fig. 2). To further analyze E and 3a immunoreactivity patterns in a quantitative rather than qualitative manner, we utilized NaturePatternMatch (NPM) to identify E or 3a protein staining patterns between viral and transiently transfected protein expression images. NPM compared similarities (or differences) based on distinct image objects/features (keypoints) in order to match these objects/features across images. The NPM algorithm does this by keypoint matching, determined by SIFT extraction (scale-invariant feature transform), which detects and compares similar patterns across multiple images and provides a rank score based on pattern similarities (Stoddard et al. 2014; Fig. 4E, F). As a control, when comparing the same image set (iE vs. iE or i3a vs. i3a) NPM generated a rank score of 1, or highly similar. Conversely, when comparing unlike image sets (iE or i3a vs. DAPI) a rank score of 4 was computed indicating no similar patterns were found. When comparing combinations of infected and transfected E or 3a protein images, a rank score of 2 was generated, suggesting they had similar but not identical patterns regardless of protein or delivery method (Fig. 4G).

### Transfected E and 3a proteins have different localization coefficients across various cellular markers

**Fig. 5:**
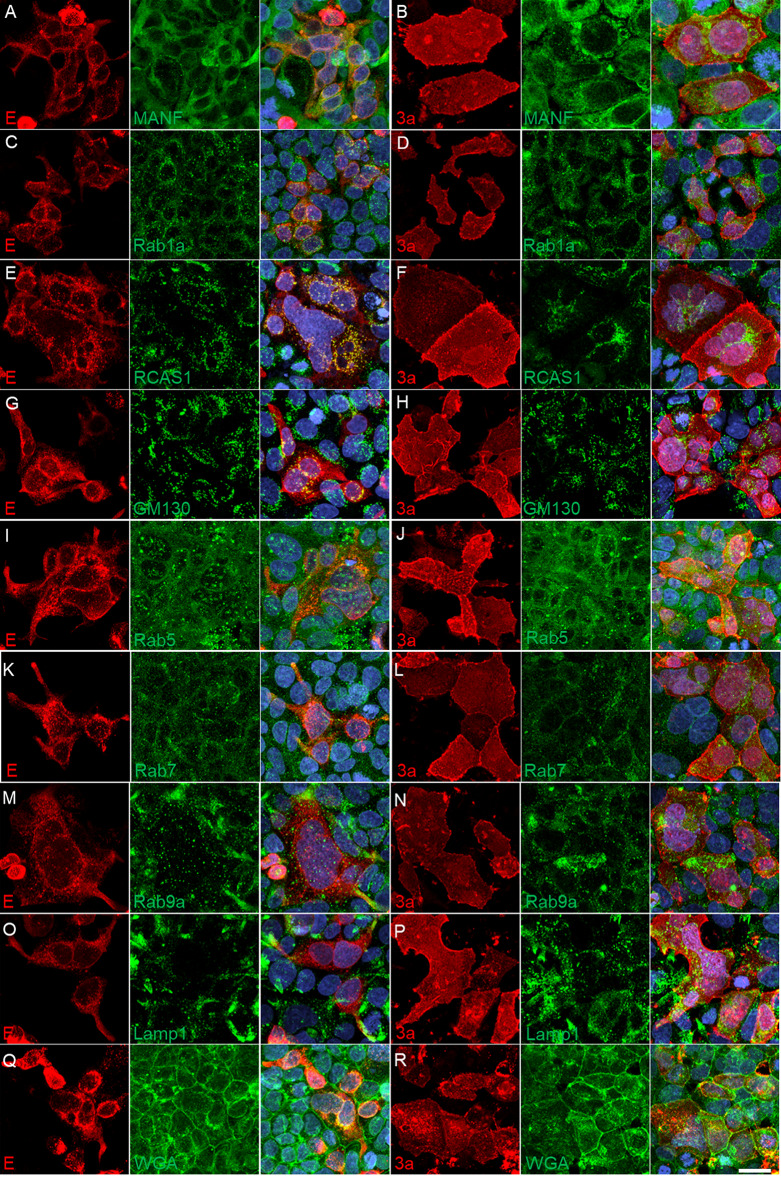
Representative confocal maximum intensity projected z-stack images of transiently transfected Caco-2 cells with SARS-CoV-2 E protein and 3a protein (2xStrep tag) plasmids. Various organelle markers were used to co-stain with SARS-CoV-2 transfected plasmid E-2xStrep or 3a-2xStrep staining to determine subcellular expression along the secretory pathway (**A**-**R**). Scale bar: 20 μm

**Fig. 6:**
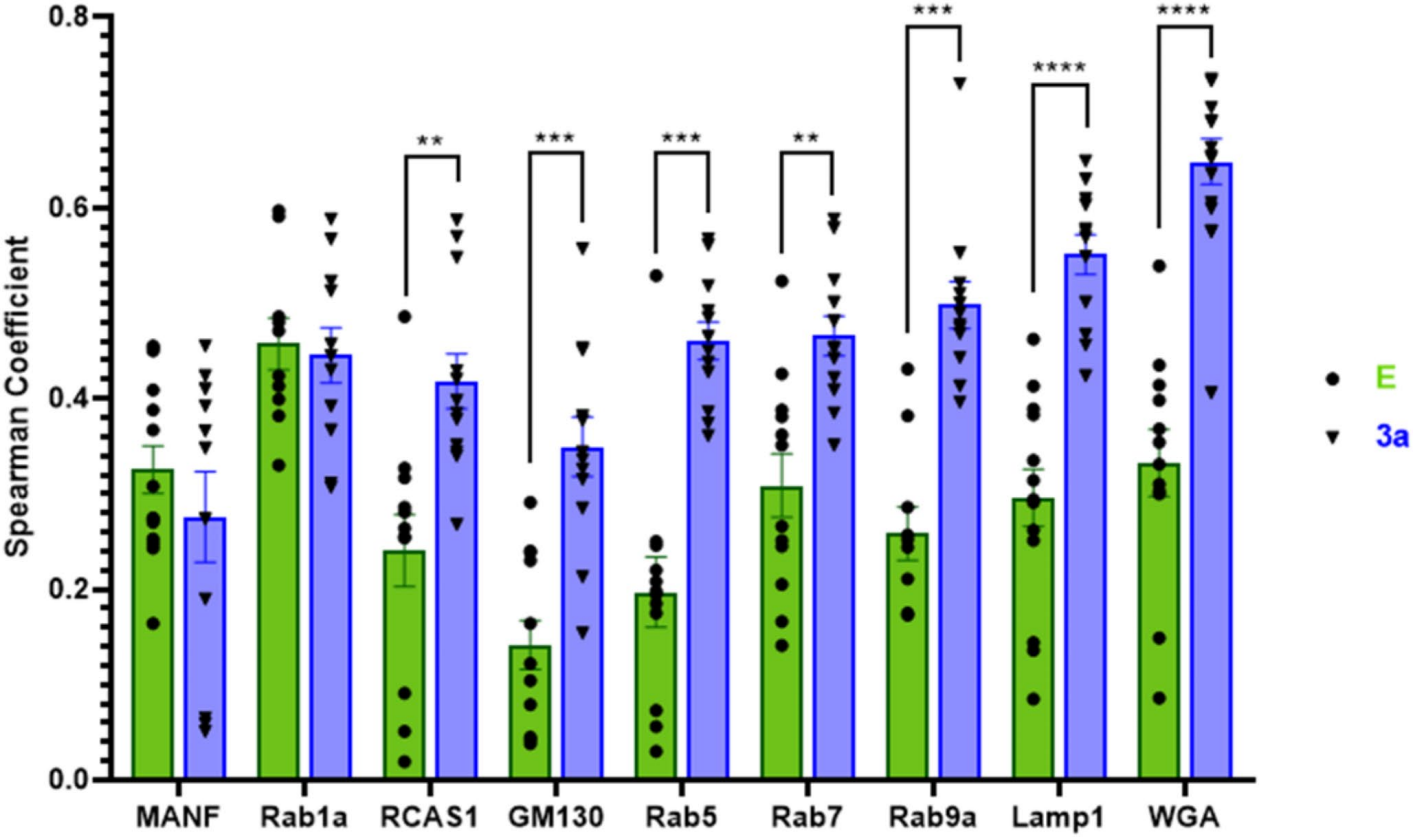
Transfected E and 3a localization with secretory pathway organelle markers. Volumetric (3D) localization was quantified in Huygen’s Pro software and the Spearman Correlation coefficient was graphed based on z-stack image ROI localization of E or 3a with cellular markers. Unpaired, two-tailed t-test comparison for each marker; ** *p* < 0.01, *** *p* < 0.001, **** *p* < 0.0001

To further identify the subcellular localization of E and 3a proteins, additional cellular protein markers were colabeled by immunofluorescence. The Spearman Correlation Coefficients were calculated between each cellular marker and E or 3a protein: MANF (ER, Spearman Coefficient mean: E – 0.33, 3a – 0.28), Rab1a (ERGIC: E – 0.46, 3a – 0.45), RCAS1 (*cis*-Golgi: E – 0.24, 3a – 0.42), GM130 (*cis*-Golgi: E – 0.14, 3a – 0.36), Rab5 (early endosome: E – 0.20, 3a – 0.46), Rab7 (late endosome: E – 0.31, 3a – 0.47), Rab9a (late endosome: E – 0.26, 3a – 0.48), Lamp1 (lysosome: E – 0.30, 3a – 0.55), and WGA (plasma membrane: E – 0.33, 3a – 0.65). Both E and 3a protein immunofluorescence overlapped with all markers; however, when comparing correlation coefficients, 3a protein localized more predominantly with GM130, RCAS1, Rab5, Rab7, Rab9a, Lamp1, and WGA while E and 3a protein correlated with MANF and Rab1a similarly (images – Fig. 5; quantification – Fig. 6).

### SARS-CoV-2 USA-WA1/2020 infection or transfected E and 3a proteins differentially modulate ER stress

**Fig. 7:**
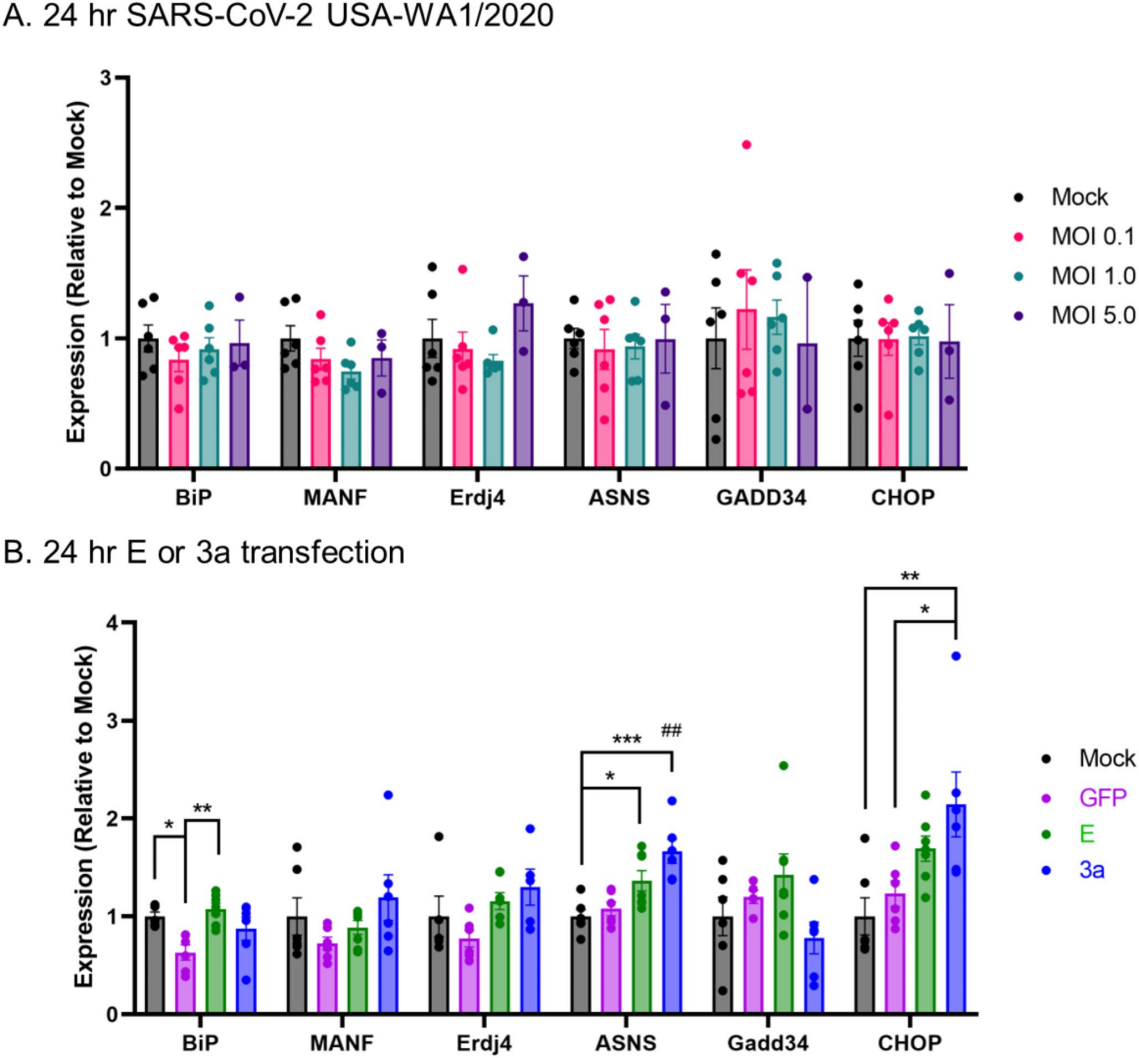
Relative gene expression levels following SARS-CoV-2 infection or protein transfection. Unfolded Protein Response expression of (**A**) SARS-CoV-2 USA-WA1/2020 infection at multiple MOIs (ddPCR) and (**B**) E- or 3a-2xStrepII transfections (dPCR) in Caco-2 cells 24 h post-delivery. Mock demonstrates a control treatment lacking (**A**) virus or (**B**) plasmid but contains all other reagent components. All gene expression values were normalized to the geometric mean of reference genes (Ube2i and GAPDH). Each gene set was compared with one-way ANOVA with Holm-Šídák’s multiple comparisons test; * *p* < 0.05, ** *p* < 0.01, *** *p* < 0.001. ## *p* < 0.01, ASNS: 3a vs. GFP

Based on E and 3a protein localization in the ER, ERGIC, and Golgi, ER stress and UPR markers were quantified 24 h post SARS-CoV-2 Washington infection or 24 h post-transfection with E and 3a expression plasmids in Caco-2 cells (Fig. 7). SARS-CoV-2 infection did not increase the gene expression of selected UPR markers at an MOI of 0.1, 1.0, or 5.0 (Fig. 7A). When transfected, E protein demonstrated a moderate increase in BiP/GRP78 (vs. GFP, *p* = 0.004) expression while 3a protein had increased gene expression of ASNS (vs. GFP, *p* = 0.0018) and CHOP (vs. GFP, *p* = 0.029; Fig. 7B). Due to the low UPR induction, thapsigargin, an inhibitor of the sarco/endoplasmic reticulum Ca²⁺ ATPase pump that causes ER calcium depletion, exodosis, and UPR activation (Trychta et al. 2018) was used to verify Caco-2 cells are susceptible to ER stress and UPR activation (Supplemental Fig. 3). ER stress and UPR markers were selected to reflect different branches and pathways involved as follows: BiP/GRP78 (immunoglobulin heavy chain binding protein) was used as a general ER stress marker (Pfaffenbach and Lee 2011); MANF (Mesencephalic astrocyte-derived neurotrophic factor) is an ER stress-responsive protein (Henderson et al. 2013); increased ERdj4 (endoplasmic reticulum DnaJ family protein) expression is indicative of IRE1 (inositol-requiring enzyme-1) activation (Cao et al. ); increased ASNS (asparagine synthetase) expression is downstream to PERK (PKR-like ER protein kinase) activation (Gjymishka et al. 2009); GADD34 (growth arrest and DNA damage-inducible protein) upregulation reflects a reversal of translation repression and suggests a recovery of protein synthesis (Marciniak et al. 2004); CHOP (CCAAT-enhancer-binding protein homologous protein) is induced by ER stress and mediates apoptosis (Oyadomari and Mori 2004).

## Discussion

### SARS-CoV-2 E and 3a proteins are conserved across multiple variants

SARS-COV-2 E protein is highly conserved across variants (USA-WA1/2020, Wuhan, Delta, and Omicron) with only a single amino acid difference in Omicron (Fig. 1A). Additionally, SARS-CoV-2 E protein is ~ 95% homologous to SARS-CoV, suggesting the E protein may have similar structure and function across variants and coronaviruses (Yoshimoto 2020; Cao et al. 2021). Similarly, SARS-CoV-2 3a protein is highly conserved across variants with two amino acid substitutions found in the Delta variant (Fig. 1A). However, SARS-CoV-2 3a protein is only ~ 72% homologous to SARS-CoV with many amino acid substitutions throughout the sequence (Yoshimoto 2020). Here, we examined both 3a and E protein from USA-WA1/2020 for their subcellular distribution and effects on the UPR pathway.

### SARS-CoV-2 E or 3a proteins exhibit similar ER and golgi localization but differ in overall staining patterning

SARS-CoV-2 E and 3a proteins localize along the secretory pathway in subcellular compartments that relate to their protein synthesis, ER retention or organelle targeting signals, and/or function. Utilizing image analysis to identify localization of E and 3a with cellular proteins, SARS-CoV-2 infected E and 3a proteins displayed similar distribution to the ER and Golgi (Fig. 2). E and 3a tagged proteins were also expressed by transfection (Fig. 3) and using the NPM method, we computationally scored infected versus transfected E and 3a protein images which confirmed similar keypoint patterns between the two methods of expressing these proteins (Fig. 4). However, the patterns were not an exact match and may reflect protein expression differences in plasmid transfection versus viral infection. Towards the latter, SARS-CoV-2 viral protein expression is modulated by the continuous interplay of viral replicative fitness and the host response.

### Transfected SARS-CoV-2 E or 3a proteins are present along the secretory pathway but have different correlative localization with subcellular markers

SARS-CoV-2 E and 3a protein, when transfected into Caco-2 cells, are expressed along the secretory pathway and localize with the ER, ERGIC, *cis-*Golgi, early and late endosomes, lysosomes, and the plasma membrane (Figs. 5 and 6). This is consistent with the literature when combining multiple studies across different cell types, tags/labels, and cellular markers (E: Boson et al. 2021, Neitthoffer et al. 2024, Henke et al. 2022, Miura et al. 2023, Miserey-Lenkei et al. 2021; 3a: Miller et al. 2023, Zhang et al. 2020, Cruz-Cosme et al. 2022, Lee et al. 2021, Miao et al. 2021); however, many of these studies did not quantify localization nor used cellular markers outside the compartments of interest leading to discrepancies where these proteins reside. Here, we utilized multiple cellular markers to demonstrate a more comprehensive mapping, both qualitative and quantitative, comparing E and 3a protein expression along the secretory pathway in Caco-2 cells.

Although E and 3a proteins were found to localize with all the employed cellular markers, the Spearman correlation coefficients between proteins were significantly different demonstrating 3a protein had a higher correlation with the Golgi, early and late endosomes, lysosome, and plasma membrane compared to the E protein (Fig. 6). However, SARS-CoV-2 infected E and 3a protein demonstrated no difference in Spearman coefficients in the Golgi (Fig. 2). While 2D, max-projected SARS-CoV-2 infected E and 3a staining patterns were mathematically similar to transfected E and 3a proteins based on extracted SIFT keypoints (Fig. 4), qualitatively, there were some visual differences that could account for the NPM score of 2 (similar) versus 1 (highly similar) and may reflect a difference in expression level and/or localization (Figs. 2 and 4).

In SARS-CoV-2 infection, protein localization is influenced by organelle retention sequences as well as viral protein interactions that aid in replication, anchoring, egress, and/or modulating host machinery (Mukherjee et al. 2020; Aoe 2020; Tebha et al. 2024). For instance, SARS-CoV-2 M protein may be responsible for the recruitment of S and E proteins to the ERGIC/Golgi (Scherer et al. 2022) and the interaction of M and E proteins modulates S protein localization and maturation allowing for viral particle assembly and release (Boson et al. 2021). Additionally, E protein expression reduced the kinetics of VSV-Gts (a viral glycoprotein commonly used as a reporter protein of cargo trafficking) and overall surface expression suggesting that E protein can alter the secretory pathway by modulating protein retention (Boson et al. 2021). SARS-CoV 3a protein was found to interact with S, M, and E proteins; however, these interactions have not been confirmed for SARS-CoV-2 (Zhang et al. 2022). Additionally, although transfected protein sequences were matched to SARS-CoV-2 USA-WA1/2020, the additional 2xStrep tag on the C-terminus may affect localization and functional interactions. SARS-CoV and -CoV-2 E proteins contain a C-terminal PDZ-binding motif sequence that when mutated or deleted (or potentially blocked by a tag) can alter interactions with host PDZ domain proteins and affect E protein function (Miura et al. 2023). SARS-CoV-2 3a protein also has important C-terminal motifs that block the fusion of autophagosomes with lysosomes to reduce viral degradation and promote viral egress (Miao et al. 2021; Zhang et al. 2021, 2024). Interestingly, SARS-CoV 3a protein does not modulate autophagy nor promote lysosomal exocytosis-mediated egress (Chen et al. 2021) further suggesting SARS-CoV and -CoV-2 3a proteins have different functions. Overall, it is important to consider that appending a tag to E and 3a proteins may alter the steady state localization when compared to wild-type infected protein.

The subcellular localization of E and 3a described herein was based on a single type of cell. Caco-2 cells are an intestinal epithelial cell line that are permissive to SARS-CoV-2, highly supportive of replication, and representative of one of the primary anatomical sites of viral RNA in humans (Wu et al. 2020). Subcellular structures such as ER and Golgi can vary by cell type and the distribution of the E and 3a proteins may vary among other cell types. The findings presented here may not generalize to all cell types but could be used as a reference when examining E and 3a protein expression in other cells.

### SARS-CoV-2 infection or transfected E and 3a proteins differentially modulate ER stress

Viruses interact with and regulate host defense mechanisms and may directly modulate UPR activation in a constant contest of pro-viral and anti-viral responses to favor viral replication or viral clearance (Byun et al. 2014; Jheng et al. 2014). As disruptions in the proteostasis network accumulate and viral protein synthesis increases, rising levels of unfolded or misfolded proteins cause ER-resident chaperone BiP/GRP78 to dissociate from ER-membrane proximal sensors and BiP-mediated activation of the UPR branches: PERK, IRE1, and ATF6 (activating transcriptional factor-6). These branches trigger a series of signal transduction and transcriptional pathways that attempt to attenuate ER stress by decreasing protein translation, increasing degradation of misfolded proteins, and upregulating production of chaperones to aid in proper protein folding. However, when acute ER stress persists the UPR can progress to apoptosis (Ghemrawi and Khair 2020; Ren et al. 2020).

Due to the localization of SARS-CoV-2 E and 3a proteins in the ER, ERGIC, and Golgi, as well as E protein’s function as a viroporin (Breitinger et al. 2023; Harrison et al. 2022), gene expression levels were measured for several ER stress and UPR genes following a SARS-CoV-2 infection (Fig. 7A) or transfection (Fig. 7B). SARS-CoV-2 infection did not increase the selected markers of ER stress or UPR in Caco-2 cells at any MOI (Fig. 7A). In other cell lines susceptible to SARS-COV-2 infection, an increase in ER stress and UPR genes were observed (Ren et al. 2020; Echavarria-Consuegra et al. 2021; Zhang et al. 2021; Shaban et al. 2021; Baral et al. 2023). Caco-2 cells are susceptible to Brefeldin A and Tunicamycin-mediated ER stress (Crespo et al. 2012) as well as thapsigargin (Supplemental Fig. 3) demonstrating that Caco-2 cells undergo ER stress and UPR activation but SARS-CoV-2 infection at the MOIs tested were not sufficient to elicit a response at 24 h post-infection. These results suggest that the degree of SARS-CoV-2-mediated ER stress and UPR in vitro is likely dependent on experimental conditions: cell line used, SARS-CoV-2 variant, MOI, and time of measurements post-infection.

Transfected cells expressing E and 3a proteins exhibited a modest increase in ER stress markers; specifically, E protein increased BiP expression while 3a protein increased ASNS and CHOP expression (Fig. 7B). The modest increases observed in UPR and ER stress genes via transfected E and 3a protein, when compared to a lack of upregulation in SARS-CoV-2 infection, may reflect plasmid protein overexpression that lacks viral expression control and/or the inability to modulate host response pathways in the absence of additional viral protein interactions.

As a pH modulating viroporin, the E protein can alkalinize organellar compartments to favor pH for viral production (Medeiros-Silva et al. 2022, 2023; Wang et al. 2023; Miura et al. 2023). Proton efflux, or alkalinization of the ERGIC, Golgi, and lysosomal lumen, is important for stabilizing viral proteins, preventing premature acid-sensitive activation of viral proteins, and inhibiting viral degradation by reducing host enzyme stability and activity in lysosomes (Nieto-Torres et al. 2015; Ghosh et al. 2020). Although the viroporin status of viral 3a protein is questionable (McClenaghan et al. 2020; Harrison et al. 2022; Miller et al. 2023), it likely plays a major role in autophagy, ER stress, and apoptosis depending on its localization. AAV transduction of 3a protein in mouse brain resulted in severe weight loss, neurological impairment, and inflammation that was likely due to a disruption in the autophagosome-lysosome pathway and accumulation of autophagosomes (Zhu et al. 2023). In vitro studies found that ER-localized SARS-CoV-2 3a protein correlated with increased ERphagy and ER stress, as well as NF-kB activation, subsequent pro-inflammatory signaling (Xu et al. 2022; Guarnieri et al. 2023; Zhang et al. 2024), and caspase3-mediated apoptosis (Ren et al. 2020). Additionally, 3a-induced ERphagy relocates the 3a protein from the ER to the autophagosome where it can directly inhibit the fusion of autophagosomes with lysosomes (Miao et al. 2021; Zhang et al. 2021) and possibly cooperating with the alkalinizing E protein, promotes viral lysosomal exocytosis-mediated egress (Chen et al. 2021).

We have provided a detailed subcellular distribution of SARS-CoV-2 E and 3a protein with emphasis on compartments associated with protein synthesis and the secretory pathway. These findings may aid in the understanding of how these proteins localize and alter host functions associated with SARS-CoV-2 infection.

## Materials and methods

### General BSL3 statement

All work involving infectious SARS-CoV-2 was approved by the Institutional Biosafety Committee (IBC) and performed in the Biosafety Level 3 (BSL3) core at the University of Rochester.

### Virus and viral infection

Vero-E6 cells (ATCC, #CRL-1586) were cultured in Eagle’s Minimum Essential Medium (EMEM; ATCC, #30–2003) supplemented with 10% (vol/vol) fetal bovine serum (FBS; Gibco, #10082147), and 1% penicillin-streptomycin (Life Technologies, #15140122) at 37 °C in a 5% (vol/vol) CO_2_ atmosphere.

SARS-CoV-2 USA-WA1/2020 was previously isolated from an oropharyngeal swab from a patient with a respiratory illness who returned from the affected region of China and was obtained through BEI Resources (#NR-52281). Viral stocks of SARS-CoV-2 were propagated in Vero-E6 cells (ATCC, #CRL-1586) at an MOI of 0.1 in EMEM (ATCC, #30–2003) supplemented with 2% (vol/vol) FBS (Gibco, #10082147), and 1% penicillin-streptomycin (Life Technologies, #15140122) at 37 °C. Viral stock titers were determined by TCID50 analysis of Vero-E6 cells four days post-infection using the Spearman-Kärber method. Viral stocks were stored at -80 °C until used in experiments.

Caco-2 BBE cells (ATCC, #CRL-2102; human colorectal adenocarcinoma epithelial cells) were cultured in Dulbecco’s modified Eagle serum (Gibco, #11965092) supplemented with 10% (vol/vol) FBS (Gibco, #10082147), 1% penicillin-streptomycin (Life Technologies, #15140122), and 1X non-essential amino acids (Gibco, #11140050) at 37 °C in a 5% (vol/vol) CO_2_ atmosphere. Caco-2 cells were selected due to their susceptibility to SARS-CoV-2 infection (Chu et al. 2020; Guo et al. 2021).

Caco-2 cells were plated at a density of 1.6e4/cm2 in a 96-well format and incubated overnight. Cells were washed with Dulbecco’s Phosphate Buffered Saline (PBS; Gibco, #14190) and incubated with infection medium (DMEM; Gibco, #11965092) supplemented with 2% (vol/vol) FBS (Gibco, #10082147), 1% penicillin-streptomycin (Life Technologies, #15140122), and 1X non-essential amino acids (Gibco, #11140050) only (mock) or containing SARS-CoV-2 (MOI of 1 or 5) for 1 h. After the incubation period, the media in all treatment groups was replaced with fresh infection medium and incubated for 8 h before downstream processing.

### Vector construction

All polymerase chain reactions were performed using 2X HotStart Q5 master mix (New England Biolabs). All constructs were made using ligation-independent cloning (In-Fusion, Takara), and transformed into a recombination-deficient bacterial strain (NEB Stable competent cells, New England Biolabs). Insert containing clones were confirmed by restriction digest analysis and DNA sequencing.

pAAV EF1a CoV2 Orf3a-2xStrep (Addgene, #213542) was constructed by inserting a codon optimized SARS-CoV-2 Orf3a-2xstrep (amplified from Addgene, #141383) into a pAAV EF1a backbone. The Addgene plasmid#141383 pLVX-EF1alpha-SARS-CoV-2-orf3a-2xStrep-IRES-Puro was a gift from Nevan Krogan (http://n2t.net/addgene:141383; RRID: Addgene_141383).

pAAV EF1a CoV2 protE − 2xStrep (Addgene, #213543) was constructed by inserting a codon optimized SARS-CoV-2 E-2xstrep (amplified from Addgene 141385) into a pAAV EF1a backbone. pLVX-EF1alpha-SARS-CoV-2-E-2xStrep-IRES-Puro was a gift from Nevan Krogan (Addgene, #141385; http://n2t.net/addgene:141385; RRID: Addgene_141385).

Both 3a and E were amplified using following primers:

Forward 5’-CGAAGTTATCGCTAGC TCGTGAGGATCTATTTCCGGTGAATTC-3’.

Reverse 5’-GAAGTTATGGCGCGCC TTACTTTTCAAACTGCGGATGTGACCATG-3’.

### ddPCR

Twenty-four hours post-infection, Caco-2 RNA was extracted using the Direct-zol RNA Miniprep Plus (Zymo, R2073) according to the manufacturer’s protocol, including on-column DNase 1 treatment. RNA quality was determined using the Bioanalyzer 2100 (Agilent, #G2939B) and RNA 6000 Nano Reagent Kit (Agilent, #50671511) with RNA Nano Chip (Agilent, #50671511) according to Bioanalyzer instructions. cDNA was made using the iScript cDNA Synthesis kit (BioRad, #1708891) according to kit instructions. Using the T100 Thermal Cycler (Bio-Rad, #1861096), the sample was primed at 25 °C for 5 min, and allowed to reverse transcribe at 46 °C for 20 min. The RT was inactivated by heating to 95 °C for 1 min, and the cycler was cooled to 4 °C. cDNA from Caco-2 cells infected with SARS-CoV-2 USA-WA1/2020 was diluted 1:5 in DNase-free water and 2.5 µL was added to 22.5 µL reaction mix consisting of 2X ddPCR Supermix for Probes (no dUTP) (BioRad), 450 nM primers, and 125nM probe for reactions using TaqMan Gene Expression Assays or 100 nM probe for reactions using probes previously reported (Trychta et al. 2018; Henderson et al. 2021). Samples were partitioned into droplets using an QX100 Automated Droplet Generator (BioRad) followed by qPCR using T100 Thermal Cycler (BioRad) with 40 amplification cycles (94 °C for 30 s, 60 °C for 1 min, repeat 39X, followed by 98 °C for 10 min, and 12 °C constant). Droplets were read using QX200 Droplet Reader and values were normalized to the geometric mean of reference genes human ubiquitin-conjugating enzyme 2i (Ube2i) and human glyceraldehyde 3-phosphate dehydrogenase (GAPDH). Data are presented as gene expression relative to mock transduction. TaqMan Gene Expression Assays included GADD34/PPP1r15A (Assay ID# Hs00169585_m1; FAM-MGB), MANF (Assay ID# Hs00180640_m1; VIC-MGB), and CHOP/DDIT3 (Assay ID# Hs01090850_m1; VIC-MGB). Remaining primer and probe sets were either labeled with FAM/BHQ1 (BiP, Erdj4, and ASNS) or HEX/BHQ1 (Ube2i and GAPDH). Apart from GAPDH (see Supplemental Table 1 for primers/probes), nucleotide and respective accession numbers have been previously reported (Trychta et al. 2018). Viral gene expression data (Supplemental Fig. 3A) was generated using the CDC 2019-Novel Coronavirus (2019-nCoV) RT PCR Diagnostic panel (2019-nCoVEUA-01) for N protein epitopes N1 and N2 expression levels.

### Plasmid transfections

Caco-2 cells were plated for 24 h (Confocal: 3e4 cells/well, 96-well glass bottom, Cellvis P96-1.5 H-N; dPCR: 5e4 cells/well, 24 well plate, Corning Falcon, #353047) and forward transfected with Lipofectamine 3000 (Invitrogen, #L3000-015) using 0.1 µg (96 well plate, imaging) or 0.5 µg (24 well plate, dPCR) DNA mixture of pAAV EF1a SARS-CoV-2 protE-2xStrep or SARS-CoV-2 3a-2xStrep added to each well for 24 h.

### dPCR

RNA was isolated from Caco-2 cells 24 h post-transfection using the Nucleospin RNA Plus kit (Machery-Negel, #740984) and performed according to the kit instructions. cDNA was diluted 1:10 in RNAse free water to prepare samples for dPCR. The reaction mixture (0.8 μm primers, 0.4 μm probes, and 4X Probe Mix (Qiagen, #250102)) was run on the QiAcuity Four Platform dPCR system (Qiagen, #911042) in a QiAcuity 8.5k 96 well Nanoplate (Qiagen, #250011). Samples were primed according to the QIAGEN standard priming profile, then cycled once at 95 °C for 2 min, followed by 40 cycles of 95 °C for 15 s and 60 °C for 30 s in sequence. See Supplemental Table 1 for primers/probes used.

### Western blot

Twenty-four hours after transfection, Caco-2 cells were lysed in 50 mM Tris-HCl (pH 7.4), 0.25% sodium deoxycholate, 150 mM NaCl, 1 mM EDTA, 1%NP-40, protease inhibitors) and proteins were separated on a 4-12% Bis-Tris NuPage gels (Thermo Fisher Scientific) using MES SDS running buffer (Thermo Fisher Scientific). Proteins were transferred to PVDF membranes (Thermo Fisher Scientific) and immunoblotted with mouse anti-NWSHPQFEK Tag antibody (Genescript, #A01732) and rabbit anti-actin (Cell Signaling, #4967 S) and detected using goat anti-mouse IR680 (LI-COR Biosciences, Lincoln, NE, #926-68070) and goat anti-rabbit IR800 (LI-COR, #925-32211). Blots were scanned using an Odyssey scanner (LI-COR).

### Immunocytochemistry

Caco-2 cells were briefly washed with 1xPBS and fixed with 4% paraformaldehyde (Sigma, #441244) for 30 min. Cells were blocked for 30 min (blocking solution: 0.4% Triton-X (Sigma, #T8787), 5% normal goat serum (Gibco, #16210-064), 1x PBS) and stained with primary antibody in blocking solution for 24 h at 4 °C and secondary antibody in blocking solution for 2 h at room temperature. Cells were then incubated with DAPI (Invitrogen, #D3571, 1:1000) for 8 min. (See Supplemental Table 1 for detailed primary and secondary antibody information).

### Confocal imaging and image analysis

Stained cells were imaged using an inverted Nikon Ti2-E A1R HD Laser Scanning Confocal Microscope with a Nikon Apochromat Lambda LWD 40x/1.15 Water objective (MR077400; Nikon, Tokyo, Japan). Images were acquired at 1024 × 1024 pixels, 0.10 μm pixel size, and 0.25 μm z-steps. Following acquisition images were denoised in Nikon Elements software using the built in denoise. AIS plugin and deconvolved using Huygens Professional using “Standard” with the Express Profile with Acuity set to -5.3 (v23.04; Scientific Volume Imaging, Netherlands). Z-stack images were then imported into Huygens Professional (v23.04; Scientific Volume Imaging, Netherlands) to quantify Spearman Correlation coefficients for FITC and TRITC channel localization based on manually drawn ROIs (3D localization). Manually drawn ROIs included only infected or transfected cells from the whole image with minimal inclusion of background (i.e. for Spearman coefficient graphs, each data point is the correlation per image and not a single cell). This also improved coefficient value accuracy as analysis was restricted to cells without including the background or thresholding (Adler and Parmryd 2010).

### NaturePatternMatch (NPM)

Denoised, deconvolved confocal images were max-projected in FIJI, saved as tiffs, and image sets were run through the NPM algorithm using the detailed instructions found in the original publication’s Supplementary Information file (Stoddard 2014). Briefly, the first algorithm extracts SIFT keypoints, or local features, from the image that are location, scale, and rotationally invariant (Lowe 1999). Due to the heterogeneity in cell morphology and overall patterning for each protein “dataset self-similarity mode” was used to compare similarity scores between whole sets of images (i.e. all infected 3a images versus all transfected 3a images). The second algorithm, NPM, compares all possible keypoint pairings across two images and ranks candidate matches by highly similar (1) to not similar (4). These rank numbers were then averaged and plotted; however, in all cases the generated rank scores per compared image sets did not vary resulting in no error bars. The order of steps/choices selected for algorithms were based on previous methods (Stoddard et al. 2014): no ROI selected (use whole image); no image enhancement performed; extract SIFT features; match SIFT feature type; texture based; bi-directional symmetric (min score); score combination, mean.

### Statistical analysis

Data was graphed in Prism v9.3.0 software (GraphPad, San Diego, CA) and analyzed using Student’s t-test or one-way ANOVA with Holm-Šídák’s multiple comparisons test; *p* = 0.05 was considered significant.

## Electronic supplementary material

Below is the link to the electronic supplementary material.

Supplementary Material 1

## Data Availability

No datasets were generated or analysed during the current study.
