# Supplementary material for: Subcellular localization of SARS-CoV-2 E and 3a proteins along the secretory pathway
Source: J Mol Histol. 2025 Mar 1;56(2):98. doi: 10.1007/s10735-025-10375-w (PMC11872775; doi:10.1007/s10735-025-10375-w)
Supplement: Supplementary file 1 — Supplementary Material 1 [file 10735_2025_10375_MOESM1_ESM.docx]

**
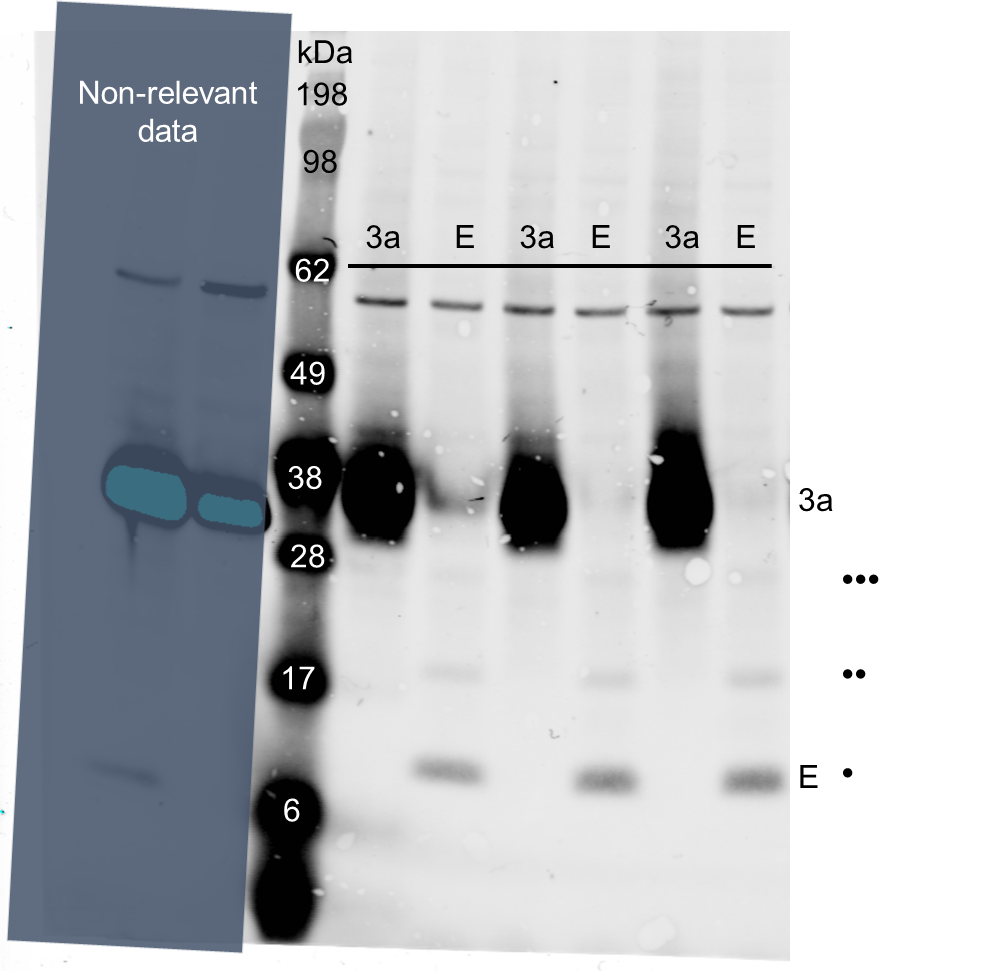
**

**Supplemental Fig. 1:** **Western blot of E-2xStrep and 3a-2xStrep transfected Caco-2 cells.** Black and white, uncropped, original western blot for Fig. 3C with anti-Strep antibody. •, ••, ••• demonstrates E protein multimer bands similar to staining patterns shown by Breitinger et al. (2023) with an N-terminus Myc tag E protein construct.

**
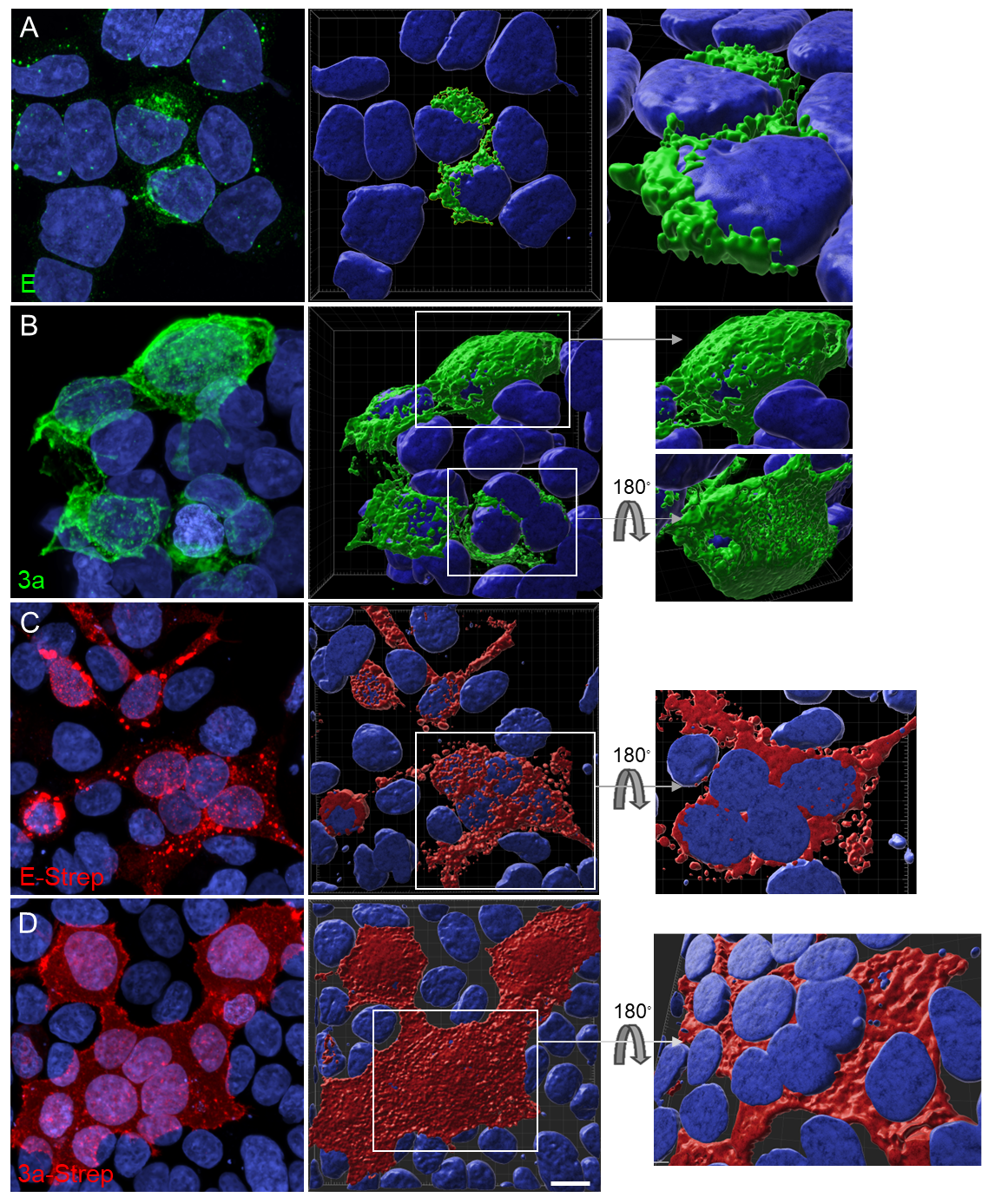
**

**Supplemental Fig. 2:** Additional qualitative observations (volumetric) of SARS-CoV-2 infected A) E and B) 3a proteins and transfected C) E protein and D) 3a protein reconstructed in Imaris software (v10.0; second and third columns). Volumetric reconstruction of 3a protein displayed a more encompassing, plasma membrane-like pattern laying on top of the nucleus where E protein was found more internal, next to and around the nucleus. Scale bar = 10um.

**
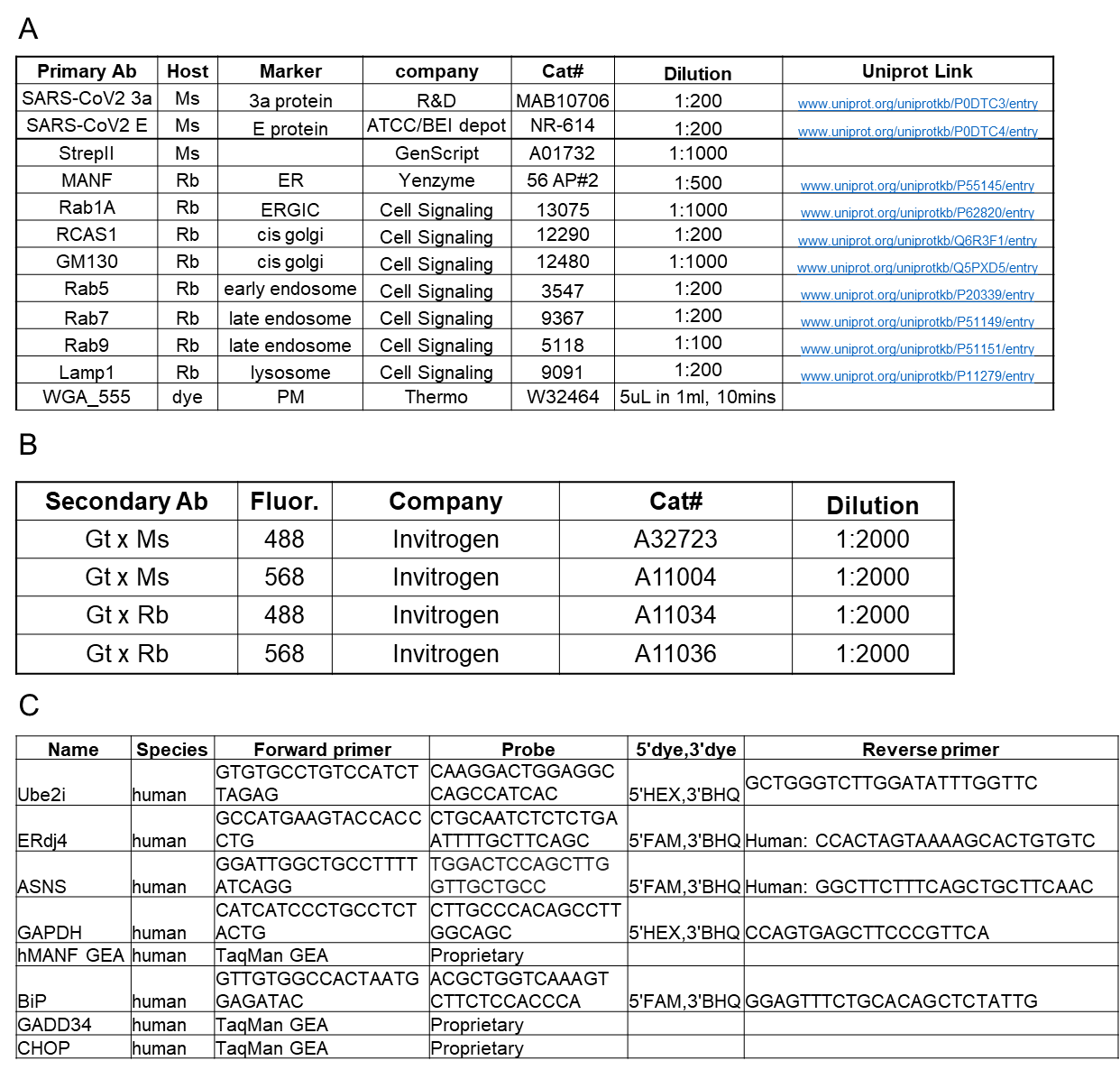
**

**Supplemental Table 1:** A) Primary antibodies, B) secondary antibodies, and C) PCR primers/probes used with relevant information.

**
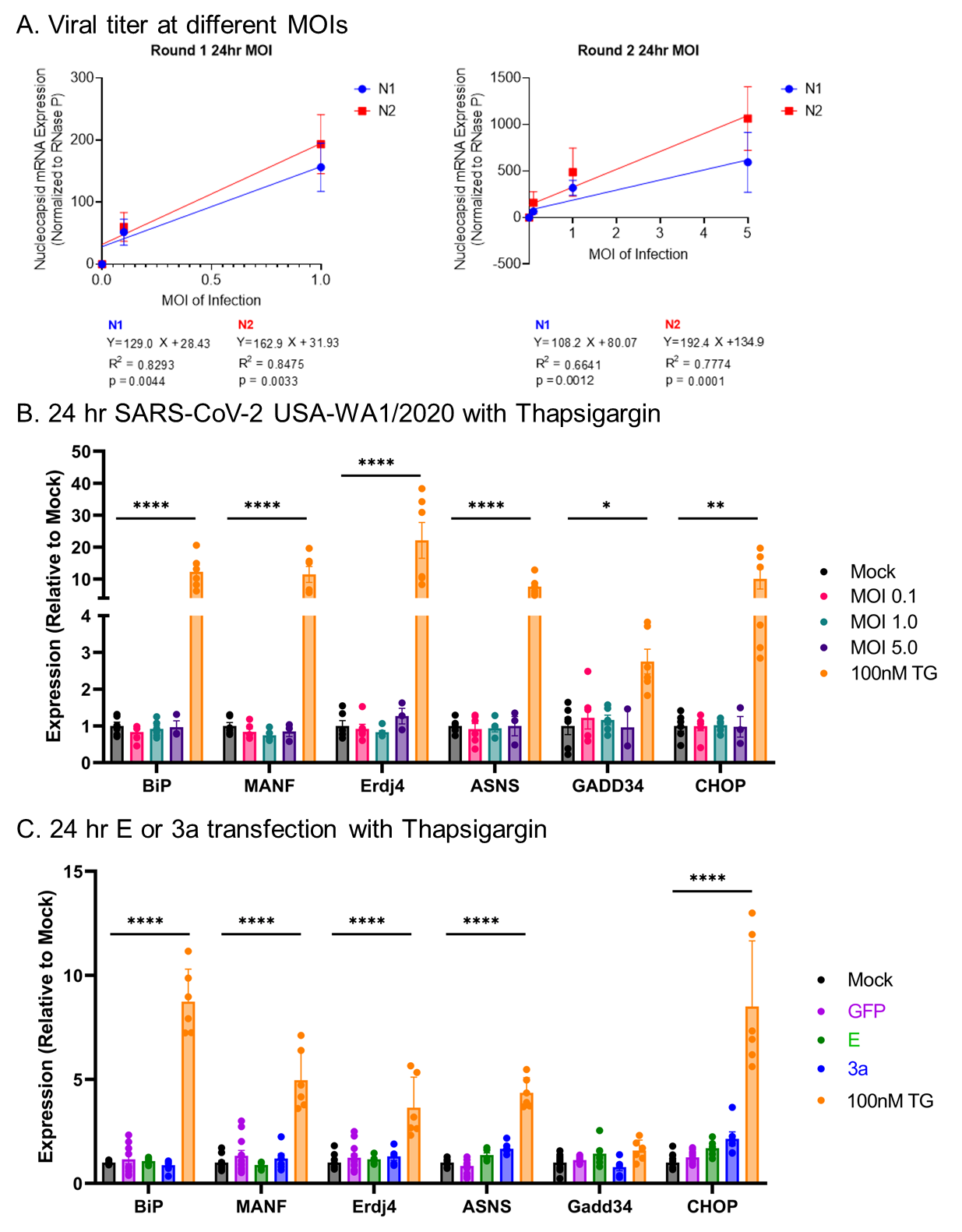
**

**Supplemental Fig. 3: Viral gene expression and relative gene expression levels following SARS-CoV-2 infection with thapsigargin.** A) Viral gene expression was generated using the CDC 2019-Novel Coronavirus (2019-nCoV) RT PCR Diagnostic panel to demonstrate Nucleocapsid expression levels via ddPCR. Caco-2 Unfolded Protein Response expression levels 24 hr post B) SARS-CoV-2 USA-WA1/2020 infection at multiple MOIs (ddPCR) and C) GFP, E, or 3a transfections (dPCR) with a mock treatment plus 100nM thapsigargin (TG) as a positive control. Each gene set was compared with one-way ANOVA with Holm-Sidak multiple comparisons test; * p < 0.05, ** p < 0.01, **** p < 0.0001.
